# Efficacy and safety of ciprofol for agitation and delirium in the ICU: A multicenter, single-blind, 3-arm parallel randomized controlled trial study protocol

**DOI:** 10.3389/fmed.2022.1024762

**Published:** 2023-01-09

**Authors:** Guo Liang Liu, Guo Zhi Wu, Dong Ge, Heng Jie Zhou, Song Cui, Kai Gao, Wei Jia Sun, Dong Hai Yu, Si Bo Liu, Jin Jie Liu

**Affiliations:** ^1^Intensive Care Unit, Dalian Municipal Central Hospital Affiliated Dalian University of Technology, Dalian, China; ^2^Department of Pharmacy, Dalian Municipal Central Hospital Affiliated Dalian University of Technology, Dalian, China; ^3^Department of Anesthesiology, Dalian Municipal Central Hospital Affiliated Dalian University of Technology, Dalian, China; ^4^Intensive Care Unit, Beijing Friendship Hospital Affiliated Capital Medical University, Beijing, China; ^5^Department of NO.2 General Medicine, Dalian Municipal Central Hospital Affiliated Dalian University of Technology, Dalian, China; ^6^Neurological Intensive Care Unit, Beijing Tiantan Hospital Affiliated Capital Medical University, Beijing, China

**Keywords:** ciprofol, agitation, delirium, intensive care unit, sedation

## Abstract

**Background:**

Agitation is very common in the intensive care unit (ICU). The causes include pain, delirium, underlying disease, withdrawal syndrome, and some drug treatments. The practical goal of ICU treatment is to find an appropriate sedation regimen to reduce pain, restlessness, and delirium. Previous trials have examined the use of dexmedetomidine, but no trials have evaluated the efficacy and safety of ciprofol, a new sedative drug.

**Methods:**

This study was a multicenter, single-blind, 3-arm parallel randomized controlled trial. ICU patients aged ≥ 18 years with agitation and delirium who met the eligibility criteria were included. The main outcome was the proportion of patients who needed additional study medication or midazolam due to agitation within 4 h after the first intravenous injection of the study medication. The secondary outcomes included the pass rate as indicated by a Richmond Agitation-Sedation Scale (RASS) score < +1, the effectiveness rate of improving delirium symptoms, the number of recurrences of agitation within 24 h, the incidence of rescue treatment, the dose and cost of analgesic and sedative drugs, the length and cost of ICU stay, and the 30-day survival period. The safety evaluation included the incidence of adverse events (hypotension, bradycardia, hypoxia, etc.) and the rate of endotracheal intubation. The subjects were randomly assigned to receive ciprofol, dexmedetomidine, or normal saline at a ratio of 1:1:1. The rates of additional drug administration within 4 h after the first injection of the study drug in the three groups were 40, 50, and 90%, respectively. A total sample size of 81 subjects was required to reach 90% power and an α of 0.05. Considering a 20% loss rate, 102 patients were enrolled and randomly assigned to the three groups in equal proportions.

**Ethics and communication:**

This trial was approved by the Ethics Committee of Dalian Municipal Central Hospital. The communication plan includes presentations at scientific conferences, scientific publications, and presentations to the public through non-professional media.

**Clinical trial registration:**

www.ClinicalTrials.gov, identifier ChiCTR220006 2799.

## Introduction

Agitation is very common in the intensive care unit (ICU). The causes include pain, delirium, underlying disease, withdrawal syndrome, and some drug treatments. The incidence of agitation varies among ICUs, but 12–70% of critically ill patients develop agitation (1). Agitation is closely related to adverse outcomes. For example, an increased duration of mechanical ventilation and prolonged hospital stay put patients at risk of life-threatening symptoms (2). The economic impact of unplanned removal of medical devices caused by agitation in a single ICU is estimated to exceed $250,000 per year (3). The economic impact of delirium is even greater: more than 164 billion dollars annually in the USA and more than 182 billion dollars annually in 18 European countries (4). Due to the serious negative impacts of agitation and delirium on the prognosis of ICU patients and the heavy burden these conditions impose on the health system, the prevention and treatment of agitation and delirium have become urgent problems in the field of intensive care medicine (5).

The practical goal of ICU treatment is to find an appropriate sedation regimen to reduce pain, restlessness, and delirium. Ciprofol (HSK3486) is a novel 2,6-disubstituted phenol derivative that, similar to propofol, binds to γ-aminobutyric acid-α (GABAA) receptors (6). In a phase 2 study of ICU patients requiring mechanical ventilation (NCT04147416), the success rate of sedation using ciprofol was 100%, with rapid recovery, no significant accumulation, and good safety (7). However, there is no evidence for the sedative effect and safety of ciprofol for ICU patients with agitation and delirium who are not mechanically ventilated. This study aimed to confirm the efficacy and safety of ciprofol in short-term (4–24 h) shallow sedation (RASS –2 to +1) in ICU patients with agitation and delirium and followed up patients for 30 days to investigate the survival, cognition, and recurrence of delirium in these patients.

## Study design

**FIGURE 1:**
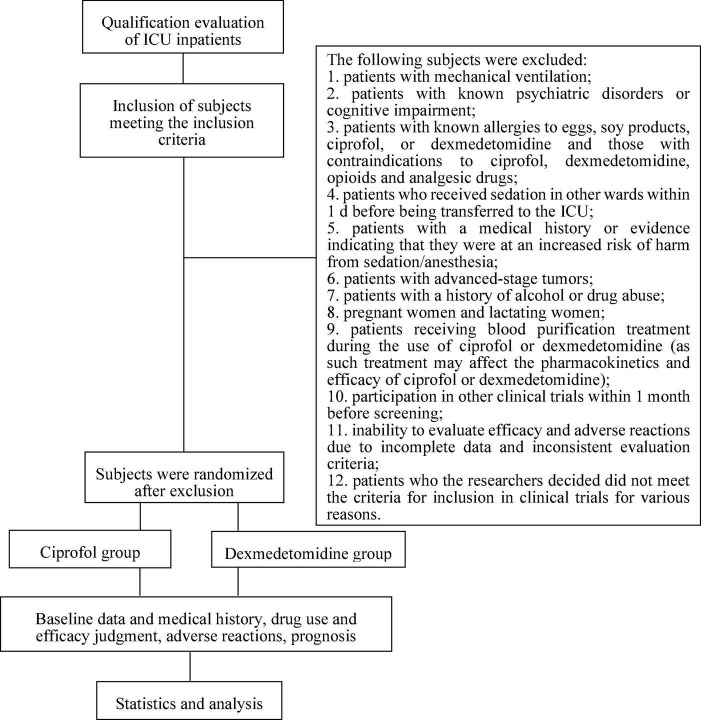
Flow chart: screening, recruitment, and grouping of patients.

This study was a multicenter, randomized, single-blind, parallel-controlled study involving 5 centers/hospitals. This trial was fully approved by the Ethics Committee of Dalian Municipal Central Hospital (20201-094-01). The trial was registered in the Chinese Clinical Trial Registry (ChiCTR2200062799)^1^ with the listed primary and secondary endpoints. The study was conducted in accordance with the clinical trial protocol (and any revisions), the Declaration of Helsinki (current revision), the *Guidelines for analgesia and sedation treatment in intensive care unit of Chinese adults*, and *Clinical Practice Guidelines for the Prevention and Management of Pain, Agitation/Sedation, Delirium, Immobility, and Sleep Disruption in Adult Patients in the ICU* (2, 8). The implementation time for the study was expected to be 2 years. The technical route is shown in Figure 1.

## Research environment

The patients were registered and treated in the ICUs of 5 centers/hospitals in China: (1) Dalian Municipal Central Hospital Affiliated Dalian University of Technology, (2) Beijing Friendship Hospital Affiliated Capital Medical University, (3) The Second Hospital of Dalian Medical University, (4) Jinzhou Municipal First People’s Hospital, and (5) Central Hospital of Zhuanghe City.

## Patient selection

We used detailed inclusion and exclusion criteria consistent with those described in a previous research report (9). Based on the inclusion and exclusion criteria, patients were enrolled and randomly assigned to receive continuous intravenous sedation with ciprofol, dexmedetomidine, or normal saline.

The inclusion criteria were as follows: (1) Patients with agitation or active delirium in the ICU were expected to need sedation for 4–24 h after randomization. (2) The expected sedation goal was within the range of the Richmond Agitation-Sedation Scale (RASS) (-2 to +1). (3) The age range of the patients was 18–85 years, and no sex restriction was applied. (4) The body mass index (BMI) of each patient was between 18 kg/m^2^ and 30 kg/m^2^. (5) Patients or their family members fully understood the purpose and significance of the trial, voluntarily agreed to participation within 24 h of admission to the ICU, and signed informed consent, including providing contact information.

The exclusion criteria were as follows: (1) patients with mechanical ventilation; (2) patients with known psychiatric disorders or cognitive impairment; (3) patients with known allergies to eggs, soy products, ciprofol, or dexmedetomidine and those with contraindications to ciprofol, dexmedetomidine, opioids, and analgesic drugs; (4) patients who received sedation in other ward within 1 day before being transferred to the ICU; (5) patients with a medical history or evidence indicating that they were at increased risk of harm from sedation/anesthesia; (6) patients with advanced-stage tumors; (7) patients with a history of alcohol or drug abuse; (8) pregnant women and lactating women; (9) patients receiving blood purification treatment during the use of ciprofol or dexmedetomidine (as such treatment may affect the pharmacokinetics and efficacy of ciprofol or dexmedetomidine); (10) participation in other clinical trials within 1 month before screening; (11) inability to evaluate efficacy and adverse reactions due to incomplete data and inconsistent evaluation criteria; and (12) patients who the researchers decided did not meet the criteria for inclusion in clinical trials for various reasons.

The levels of sedation and delirium were assessed using the RASS and the Confusion Assessment Method for the ICU (CAM-ICU) (10, 11). Agitation was defined as RASS ≥ +2, and active delirium was defined as CAM-ICU positive with RASS ≥ +2.

## Test group

To comprehensively determine the efficacy and safety of ciprofol, this study included a blank control group receiving normal saline and a drug control group receiving dexmedetomidine at a ratio of 1:1:1. Dexmedetomidine was selected as the control drug because it is a continuous infusion sedative drug recommended by many guidelines. In many countries, including the USA and China, it is usually used for long-term sedation in the ICU (2, 8, 12). The replacement block randomization method was used (the block size was set to 6), and the subjects were randomly assigned to receive saline, ciprofol, or dexmedetomidine at a ratio of 1:1:1.

This study was designed to be single-blind. The patients and their families and the case report form (CRF) data analysis researchers did not know the identities of the patients in the experimental groups. Because the ICU patients were critically ill, the clinicians and CRF data collection researchers could not be blinded. The clinicians were mainly responsible for deciding when to begin sedation, adjust the dose, and end sedation.

At the time of registration and after signing the consent form, detailed information about prior sedation and analgesic treatment, baseline demographics, delirium occurrence, and disease severity were recorded.

## Study drug management

Researchers confirmed that the RASS of each patient reached ≥ +2 before starting to administrate the study drug. Prior to drug administration, the CAM-ICU was used to assess the patient’s delirium status. The sedatives used before study registration were discontinued before the start of the study drugs.

In the experimental group, continuous intravenous pump injection of ciprofol was performed with a loading dose of 0.1 mg/kg and a maintenance dose of 0.05–0.8 mg/kg/h. The control group received a continuous intravenous pump injection of dexmedetomidine for sedation, with a loading dose of 0.1 mcg/kg and a maintenance dose of 0.03–0.7 mcg/kg/h. The specific study drug use is shown in Table 1.

**TABLE 1:** Administration of the study drug.

Study drug	Loading dose	Maintenance dose	Allowed top-up dose during maintenance administration
Ciprofol group	0.1 mg/kg, intravenous infusion (undiluted), administration time 30 s	Start maintenance at 0.3 mg/kg/h, dose can be up- or downregulated at 0.05–0.1 mg/kg/h; range of the maintenance dose: 0.05–0.8 mg/kg/h	0.05 mg/kg each time, each top-up should have at least a 2-min interval
Dexmedetomidine group	0.1 mcg/kg, intravenous infusion (dilute with 0.9% sodium chloride solution to a concentration of 4 mcg/ml), administration time 10 min	Start maintenance at 0.2 mcg/kg/h, dose can be up- or downregulated at 0.03–0.1 mcg/kg/h; range of the maintenance dose: 0.03–0.7 mcg/kg/h	0.1 mcg/kg each time, each top-up should have at least a 15-min interval

The RASS sedation assessment scale (RASS) was used to assess the level of sedation and to control the rate of drug administration. The sedation goal was RASS –2 to +1 points. When the RASS score exceeded the target range, the drug infusion rate was increased or decreased until the target RASS score was reached. If the sedation was too deep (RASS –3 to –5 points), the infusion of the study drug was stopped until the patient returned to the acceptable sedation range. Sedation assessment was performed at least every 4 h, and the dose of the study drug was adjusted by the clinical medical staff according to the RASS score and recorded in the nursing record.

Patients in the two groups who were not sufficiently sedated by study drug titration were given midazolam at a dose of 0.01–0.05 mg/kg. The injection time was 3 min, and the drug was administered again at 15-min intervals until sufficient sedation (RASS –2 to +1) was achieved. The maximum dose within 8 h was 4 mg. The lowest maintenance dose of the study drug was infused continuously for 4 h in patients with RASS scores < +1, which indicated that a subject no longer needed sedation, thus warranting discontinuation of study drug infusion. At this time, the CAM-ICU was used to re-evaluate the patient’s delirium.

Many guidelines highlight the importance of analgesic treatment, given that it is the basis of sedation treatment (2, 8, 13). Analgesics were used according to a standardized procedure. All subjects were monitored using the Critical-Care Pain Observation Tool (CPOT), and fentanyl analgesia was used to maintain a CPOT score of < 3 (14). Analgesia with a small dose of fentanyl (0.5–1.0 μg/kg) was performed once every 15 min as needed. Fentanyl analgesia was also given before expected harmful stimuli, such as fiberoptic bronchoscopy or arteriovenous catheterization. The use of fentanyl patches was prohibited. The use of other sedatives or analgesics was prohibited during the study. The total time of drug administration (including the loading dose and the maintenance dose) was at least 4 h ± 30 min, and the longest time was not more than 24 h ± 30 min. The study drug infusion was stopped if the investigator believed it was in the patient’s best interest to discontinue the drug.

During the drug administration process, circulatory and respiratory functions were always monitored, and airway assistance measures, artificial ventilation and other resuscitation devices were readily accessible. Symptoms before and after drug administration and their fluctuations were recorded. When adverse reactions occurred, the symptoms, drug doses, intervention measures, and medication time were recorded.

Ciprofol was acquired from Haisco Pharmaceutical Group Co., Ltd., and formulated as 2 ml:50 mg (lot number 20220302). Dexmedetomidine was obtained from the Yangtze River Pharmaceutical Group and formulated as 2 ml:200 mcg (lot number 22071431).

## Effectiveness evaluation

The primary endpoint was the proportion of patients who needed additional study medication or midazolam due to agitation within 4 h after the first intravenous injection of the study medication.

The secondary endpoints were as follows: (1) the proportion of patients who achieved a RASS score < +1 within 4 h after the first intravenous injection of the study medication; (2) the effective rate for improving delirium symptoms; (3) the number of recurrences of agitation within 24 h; (4) the proportion of patients who underwent tracheal intubation and received emergency drugs within 24 h; (5) the dose and cost of analgesic and sedative drugs; (6) the duration and cost of the ICU stay; and (7) short-term mortality of patients when followed up for 30 days.

## Safety evaluation

The incidence of adverse reactions (including respiratory depression, hypoxia, hypotension, hypertension, tachycardia and bradycardia symptoms, and elevated blood bilirubin, alanine aminotransferase, and triglyceride) and the rate of tracheal intubation during medication were evaluated. Two ICU specialists and two neurologists defined each subject’s adverse reactions in detail based on the drug instructions and the evidence reported in previous studies (7, 15).

1) Definition of absolute and relative hypotension: systolic blood pressure < 90 mmHg or a decrease of more than 20% of that before medication or diastolic blood pressure < 50 mmHg.
2) Definition of absolute and relative hypertension: systolic blood pressure > 180 mmHg or more than 20% higher than that before medication or diastolic blood pressure > 100 mmHg.
3) Definition of absolute and relative bradycardia: heart rate < 40 beats/min or more than 20% lower than that before medication.
4) Definition of absolute and relative tachycardia: heart rate > 120 beats/min or more than 20% higher than that before medication.
5) Definition of absolute and relative respiratory depression: respiratory rate < 8 breaths/min or lower than baseline by more than 25%.
6) Definition of absolute and relative hypoxia: SpO_2_ < 90% or lower than baseline by 10%.
7) Definition of elevated blood bilirubin: blood bilirubin > 25% higher than that before medication.
8) Definition of elevated alanine aminotransferase: alanine aminotransferase > 25% higher than that before medication.
9) Definition of elevated triglycerides: triglycerides > 25% higher than that before medication.

For any risks that occurred during the study, the investigator promptly provided correct and reasonable individualized medical treatment to the subjects according to the specific conditions of the subjects to protect the rights and interests of the subjects to the maximum extent. The investigators conducted follow-up surveys of all adverse events (including serious adverse events), with regular follow-up according to the disease condition until the final outcome of the adverse events. The follow-up process and the outcome of the adverse events were recorded. Emergency orotracheal intubation is indicated in any situation in which definitive control of the airway is needed. *New England Journal of Medicine* (NEJM)-recommended indications include cardiac or respiratory arrest, failure to protect the airway from aspiration, inadequate oxygenation or ventilation, and impending or existing airway obstruction (16).

## Data management and monitoring

All data were collected during the clinical trial. All raw data were recorded on the online data collection form by the appropriate researchers, and the accuracy of the data input was confirmed by two people. The data were processed in an anonymous and encrypted manner, and a limited number of people were allowed to access the data. The data were coded using the unique identification associated with the individual study participants. The decision to lock the database was made by the chief investigator, database administrator, and statistical analyst in charge of the statistical analysis. The research coordinator at each center supervised the conduct of the study. In addition, this experiment was closely monitored by a certified external auditor to ensure that the research activities were conducted in accordance with the protocol, clinical practice guidelines and applicable regulatory requirements. The data will be stored in double backup mode for at least 5 years after the end of the study.

## Study quality control and supervision

We established a quality assurance system, and a designated coordinator will guide the investigators when conducting clinical trials in accordance with the protocol, clinical practice guidelines, and applicable regulatory requirements. The coordinator was responsible for reviewing the original data records and case report forms, investigating any violations, ensuring that researchers have a detailed and accurate understanding of the research program, and assessing whether the procedures were correctly implemented. Any quality problems were relayed to the main researcher, and appropriate measures were taken immediately to solve the problems.

When the number of subjects reached half of the expected sample size, a mid-term evaluation was conducted. The drugs’ clinical index data were preliminarily analyzed, the risk-benefit relationship of the trial drugs was comprehensively weighed in terms of effectiveness and safety, and a major decision was made regarding whether to “continue the trial,” “continue the trial after adjusting the scheme,” or “terminate the trial.” If problems were found and the protocol needed to be modified or adjusted, all relevant information was submitted to the Ethics Committee for approval before implementation.

## Statistics

This study was a clinical randomized controlled trial. The three groups were a blank control group, a ciprofol group, and a dexmedetomidine group, with a ratio of 1:1:1. The rate of additional drug administration within 4 h after the first injection was the main outcome indicator. According to the pre-experiment results, the rates of additional drug administration in the three groups were 90, 40, and 50%, respectively. The type I error (false-positive) was set to 0.05, and the efficacy reached 90%. The total sample size of the three groups calculated by PASS (version 15.0.5) was *N* = 81 cases. Considering a loss rate of 20%, the total number of subjects required for the final three groups was 102, with at least 34 subjects in each group.

The analysis was performed according to intention-to-treat analysis including all randomized participants, and the analysis was performed in their randomized groups, regardless of the actual treatment received. For continuous numerical variables, the numbers, means, medians, standard deviations, minimums, maximums, and coefficients of variation (CVs, if applicable) were analyzed using the independent sample *t*-test or Wilcoxon rank sum test. Categorical variables were given as rates (percentages) and were analyzed using the Pearson *X*^2^ test or Fisher’s exact probability method. The baseline was defined as the last non-missing observation data collected before the first use of the study drug. The normality of the data was examined using the Shapiro–Wilk test and the Q-Q plot in SPSS. We used SPSS (version 26.0) for analysis. All statistical inferences were performed using two-sided tests. The statistically significant test level was set as 0.05, and the confidence interval (CI) of the parameters was estimated using the 95% CI.

### Analysis of the main effectiveness results

The purpose of this study was to evaluate the efficacy and safety of ciprofol for agitation and delirium in the ICU. The main effectiveness index was the comparison of the rate of additional drug administration within 4 h after the first injection, which corresponded to qualitative data and was analyzed using the Pearson *X*^2^ test or Fisher’s exact probability method.

### Analysis of secondary effectiveness results

Study drug use, the number of recurrences of agitation within 24 h, the dose and cost of analgesic and sedative drug application, and the length and cost of the ICU stay were assessed using the independent sample *t*-test or the Mann–Whitney test. The proportion of patients who achieved a RASS score < + 1 within 4 h after the first intravenous injection of the study medication, the effective rate for improving delirium symptoms, and the proportions of patients who received tracheal intubation or use of emergency medicine within 24 h in the two groups were compared using the *X*^2^ test.

### Safety analysis

The Pearson *X*^2^ test was used to compare the incidence of adverse events between the two groups, and the adverse events in this study were tabulated.

## Discussion

As China’s first innovative class 1 intravenous anesthetic drug, there are very few clinical trials related to ciprofol. To the best of our knowledge, this is the first clinical trial to investigate the applicability and safety of ciprofol as a continuous pump-in sedative in ICU patients with agitation and active delirium, especially in patients with non-mechanical ventilation.

Dexmedetomidine is a high selectivity α-adrenergic receptor agonist with analgesic and sedative effects. The positive effects of dexmedetomidine have been widely reported, including reducing the incidence of postoperative delirium, prolonging sleep time, delaying the occurrence of delirium, and shortening the duration of delirium in elderly patients (17–20). Although delirium is not among its indications, some domestic and foreign guidelines recommend dexmedetomidine for the treatment of delirium (13, 21). However, the adverse events of dexmedetomidine should not be ignored, which mainly include cardiovascular system reactions, respiratory system reactions, neuropsychiatric disorders, and others (22, 23). Two recent meta-analyses clearly indicate that dexmedetomidine is associated with a greater risk of bradycardia and hypotension in various ICU patients (24, 25). Considering that ICU patients often have multiple diseases and organ function damage or failure to varying degrees, ICU doctors are always concerned during the medication process. Effective and safe sedative and delirium control drugs have long been goals of ICU physicians.

Ciprofol has a shorter half-life than dexmedetomidine. The plasma concentration of ciprofol showed three-phase elimination, and the corresponding half-lives were 0.54 min (*t*_1/2_, α), 6.26 min (*t*_1/2_, β), and 105 min (*t*_1/2_, γ), respectively (26). Ciprofol is an alkyl phenolic compound. Phase II UDP-glucuronosyltransferases (UGTs) and phase I CYP2B6 are the main metabolic enzymes for ciprofol. Ciprofol is rapidly oxidized in the body or combined with glucuronic acid and sulfuric acid, and its metabolites are inactive (27). Therefore, unlike dexmedetomidine, clinicians do not have to consider the cumulative effect of sedatives.

Because ciprofol has a higher lipid solubility than propofol, the concentration of free molecules in the emulsion is significantly lower than that of propofol, which may reduce injection pain. In an experiment in which hypnosis was induced in rats and dogs, ciprofol had a higher therapeutic index than propofol. At the same dose, the hypnotic efficacy of ciprofol was approximately 4–5 times that of propofol (27). Similar results were observed in healthy subjects in phase I clinical trials (6). Compared with propofol, the average dose per hour, the average loading dose, and the average maintenance dose of ciprofol for sedation during colonoscopy were approximately fivefold lower, and the incidence of cardiovascular adverse events was lower (7). The dosage required for sedation is lower, which reduces the amount of lipid infusion, thereby reducing the adverse reactions caused by excessive lipid infusion for prolonged sedation, such as hypertriglyceridemia or propofol infusion syndrome.

In summary, these findings demonstrate that this drug has great potential as a new sedative drug in the ICU. If the drug shows the same beneficial properties in critically ill patients as in previous studies, it may become a new choice for patients and clinicians.

## Ethics statement

This trial was approved by the Ethics Committee of Dalian Municipal Central Hospital. Patients or their family members fully understood the purpose and significance of the trial, voluntarily agreed to participation within 24 h of admission to the ICU, and signed informed consent, including providing contact information.

## Author contributions

GL, GW, and DG: methodology, software, formal analysis, investigation, and writing—original draft. HZ, SC, and KG: methodology, software, formal analysis, and investigation. WS and DY: methodology, validation, data curation, and supervision. SL and JL: conceptualization, writing—reviewing and editing, supervision, project administration, and funding acquisition. All authors contributed to the article and approved the submitted version.

## Footnotes

1
http://www.chictr.org.cn/showproj.aspx?proj=174945
